# Embryonic Insight: Mouse Histology in 3-D

**DOI:** 10.1289/ehp.114-a596

**Published:** 2006-10

**Authors:** Julian Josephson

Histology, the study of how cells are organized into tissue, is a keystone of the biological sciences. For decades, histologists have depended upon methods such as the tried-and-true slides of stained tissue sections. Although much has been learned through these methods, how much more would be learned if histologists could view tissues in three dimensions?

Today this advance is becoming a reality thanks to a newly developed ability to combine ultra–high resolution/microscopic computed tomography (microCT) scans with high-tech computer protocols to produce detailed 3-D images of mouse embryos. Besides the marked advantage of a 3-D perspective, the technique also achieves improvements in resolution, time required, and costs for studying developmental patterning effects attributable to genetically engineered mutations and chemically induced embryotoxicity.

The research team that reported the development of this method, known as microCT-based virtual histology (MBVH), was led by Charles Keller, an assistant professor of cellular and structural biology at the Children’s Cancer Research Institute, University of Texas Health Sciences Center at San Antonio. The innovative technique was reported in *PLoS Genetics* in April 2006.

## A Better Mouse Sample

Mouse embryos are commonly used to study gene function. The team’s original aim was to develop a method for rapidly assessing birth defects associated with targeted disruptions of each gene in the mouse embryo. According to the authors, this called for “systematic, interdisciplinary approaches to analyzing patterning defects in the mouse embryo.”

What emerged, the authors wrote, was a “novel, rapid, and inexpensive method for obtaining high-resolution virtual histology for phenotypic assessment of mouse embryos.” Besides determining how genetic polymorphisms may contribute to end points such as birth defects and cancer, this new technique can perhaps be used to assess the safety of new medicines and other chemicals.

The traditional method used in histology is difficult and time-consuming. Mouse embryos that have genetic mutations or that have sustained damage from chemicals are killed, embedded in wax, and sliced into thin sections. Next, they are stained and placed on slides for microscopic examination. “[The new] technique allows us to get at a lot more tissues other than bone, such as internal organs, which [conventional] microCT scans of unstained tissue can’t pick up,” says coauthor Chris Johnson, a distinguished professor of computer science who directs the University of Utah’s Scientific Computing and Imaging Institute.

The technique uses a 1% solution of osmium tetroxide (OsO_4_), a major fixative of choice for electron microscopy, to stain tissues differentially. Images of whole embryos are then made by volumetric X ray microCT, with as little as 2 hours needed to achieve isometric resolutions of 27 μm, or 12 hours to achieve resolutions of 8 μm.

The X ray microCT scans of mouse embryos thus generated are converted using computer visualization techniques into detailed 3-D images that show the mouse’s exterior and interior. Instead of being physically sliced, the special dyes or fixatives permeate the skin and other membranes of a mid-gestation embryo; in older embryos and fetuses, the skin must be removed for the stain to penetrate.

## What’s in There?

Johnson and his group wrote an algorithm that distinguishes and visualizes various organs and structures in the mouse embryo based on the microCT scan data. This produces a virtual rendering of the scan data that also includes a virtual light source. The 3-D embryo image can therefore be rendered with shadows, which makes it easier for the human eye to understand and interpret the image.

The new technique furthermore allows users to create transparent images or even produce cutaways, so that internal organs and body parts become visible. Moreover, features as small as 8 μm can be observed. The purpose is to permit geneticists to examine anatomical features of large numbers of embryos, each with a different gene disabled. Then it becomes possible to observe the disruption of normal functions of many genes faster than by existing methods.

Indeed, with MBVH, scientists can examine as many as 120 mouse embryos at the same time, each with a different gene knocked out, to detect a specific defect. In addition, they can observe as many sets of 120 embryos as needed in order to detect those that require closer examination at higher resolution—all with the same equipment.

Time is saved, too. The 2 hours it took the research team to obtain scans of OsO_4_-stained embryos, with a 27-μm isometric resolution, is a vast improvement over the tedious techniques of histological sectioning of early embryos, which needs to be done with painstaking precision and takes 1 to 3 working days to complete.

Magnetic resonance microscopy has taken some of the procedural sting out of embryo sample preparation, but it still requires expensive, specialized equipment. The team was able to use off-the-shelf specimen scanners, thereby showing that substantial cost savings are possible—a total cost per scan of $1.50 per embryo versus $600.00 per embryo with magnetic resonance microscopy.

If higher resolution or increased definition becomes necessary, a stained embryo can be scanned at a resolution greater than 8 μm. Indeed, a 6-μm resolution has been achieved in the same time as the 8-μm scan using a different instrument. Keller and a colleague are now in the process of commercializing the new technique through their company Numira Biosciences.

## MBVH in Practice

For a practical example of how the technique works for high-throughput phenotyping, the researchers used transgenic mouse embryos with substantial malformations of the developing brain and upper spinal cord. Examining these malformations under the dissecting microscope would have been of little use in determining what structure was what. “Even light microscope sections were confusing,” says Keller. The microCT scans were what helped the researchers understand which part of the brain was overgrown and which was underdeveloped as a result of a *Pax3* gene mutation.

These embryos were scanned at a resolution of 27 μm, then rendered to visualize the forebrain, midbrain, and hindbrain vesicles; the liver; and the heart wall and cardiac vesicles. The complex 3-D organization of the mutated brain sections was made plainly visible. These views would have been impossible with paraffin-embedded specimen histology, according to Keller and his colleagues.

Among plausible applications for MBVH could be enhanced high-throughput analysis of possible side effects of drugs and effects of exposure to environmental contaminants in preclinical toxicology studies. Also feasible might be tumor vascular pattern analysis for biopsies of tumors in patients who undergo anti-angiogenesis therapy. Moreover, the NIH has launched a project to evolve knockout mice for each of the 25,000 mouse genes. Given the large number of samples that can be scanned simultaneously, MBVH technology could help speed up this process.

## Figures and Tables

**Figure:**
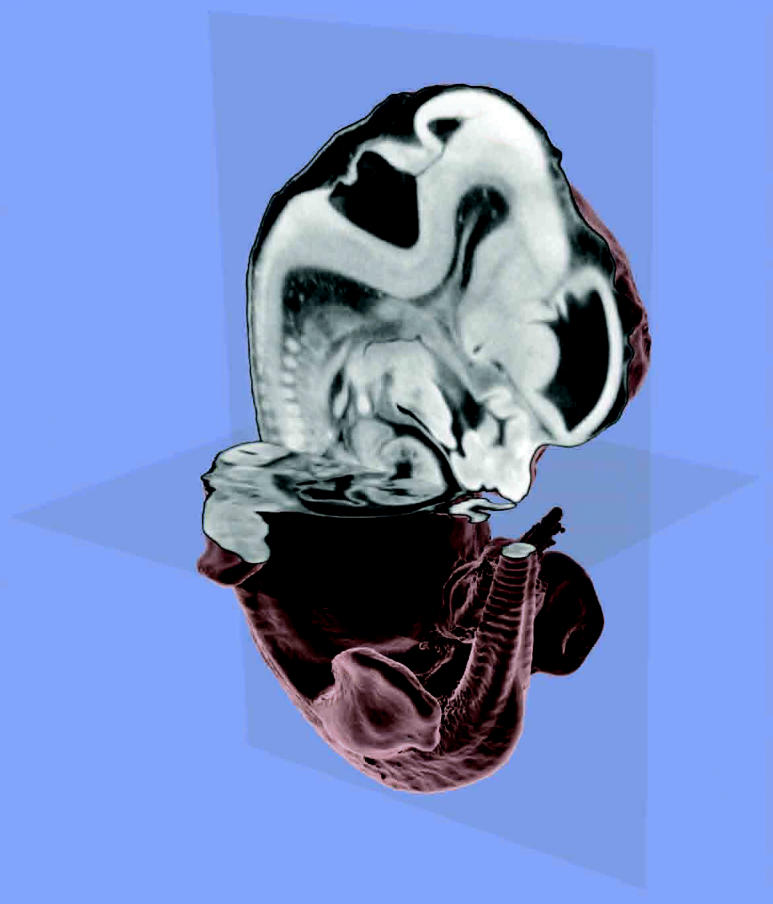


**Figure:**
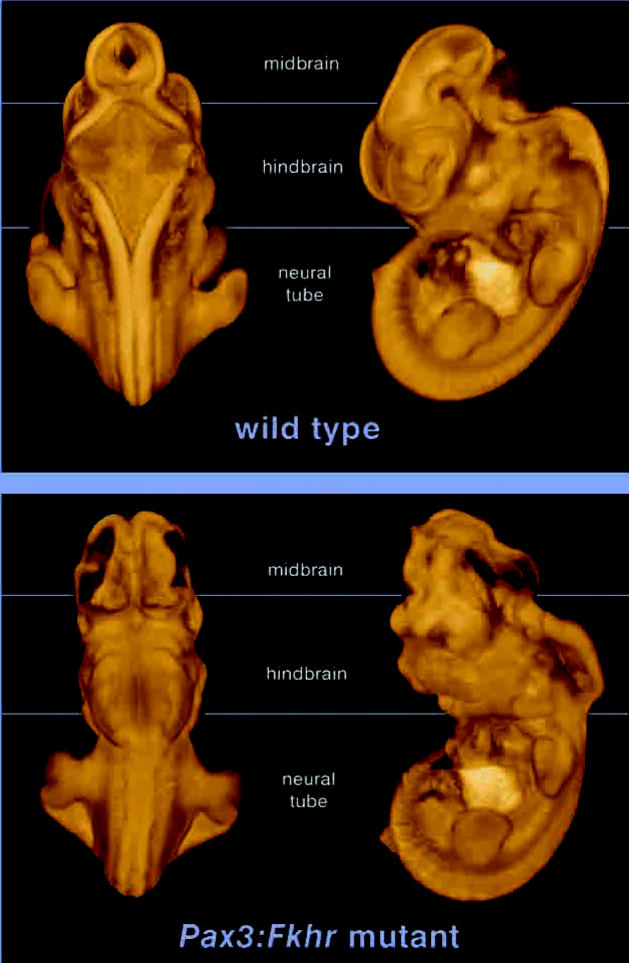
The data are in the details MicroCT scanning of normal (top) and mutated (bottom) mouse embyos allowed researchers to compare effects of the *Pax3* gene mutation on the brain and spinal cord that would have been difficult to determine with traditional histology.
